# Enhanced anti-tumor activity of a new *curcumin*-related compound against melanoma and neuroblastoma cells

**DOI:** 10.1186/1476-4598-9-137

**Published:** 2010-06-03

**Authors:** Marina Pisano, Gabriella Pagnan, Maria Antonietta Dettori, Sara Cossu, Irene Caffa, Ilaria Sassu, Laura Emionite, Davide Fabbri, Michele Cilli, Fabio Pastorino, Giuseppe Palmieri, Giovanna Delogu, Mirco Ponzoni, Carla Rozzo

**Affiliations:** 1 Istituto di Chimica Biomolecolare, CNR, Sassari, Italy; 2 Laboratory of Oncology, "G. Gaslini" Children's Hospital, Genoa, Italy; 3 Animal Research Facility, Istituto Tumori, Genoa, Italy

## Abstract

**Background:**

Sharing the common neuroectodermal origin, melanoma and neuroblastoma are tumors widely diffused among adult and children, respectively. Clinical prognosis of aggressive neuroectodermal cancers remains dismal, therefore the search for novel therapies against such tumors is warranted. *Curcumin *is a phytochemical compound widely studied for its antioxidant, anti-inflammatory and anti-cancer properties. Recently, we have synthesized and tested *in vitro *various *curcumin*-related compounds in order to select new anti-tumor agents displaying stronger and selective growth inhibition activity on neuroectodermal tumors.

**Results:**

In this work, we have demonstrated that the new α,β-unsaturated ketone D6 was more effective in inhibiting tumor cells growth when compared to *curcumin*. Normal fibroblasts proliferation was not affected by this treatment. Clonogenic assay showed a significant dose-dependent reduction in both melanoma and neuroblastoma colony formation only after D6 treatment. TUNEL assay, Annexin-V staining, caspases activation and PARP cleavage unveiled the ability of D6 to cause tumor cell death by triggering apoptosis, similarly to *curcumin*, but with a stronger and quicker extent. These apoptotic features appear to be associated with loss of mitochondrial membrane potential and cytochrome *c *release. *In vivo *anti-tumor activity of *curcumin *and D6 was surveyed using sub-cutaneous melanoma and orthotopic neuroblastoma xenograft models. D6 treated mice exhibited significantly reduced tumor growth compared to both control and *curcumin *treated ones (Melanoma: D6 *vs *control: *P < 0.001 *and D6 *vs curcumin P < 0.01; *Neuroblastoma: D6 *vs *both control and *curcumin*: *P < 0.001*).

**Conclusions:**

Our data indicate D6 as a good candidate to develop new therapies against neural crest-derived tumors.

## Background

Malignant melanoma (MM) and neuroblastoma (NB) are different cancers which share a common neuroectodermal origin, besides being dissimilar for all other pathological aspects such as tissue involvement, metastasis development and age of onset.

MM, the most lethal skin cancer, preferentially develops metastases in lymph-nodes and visceral sites (mostly lung, liver and bone-marrow): it also presents a high frequency of skin metastases. Its incidence rates have increased continuously during the last decades in fair skin populations of western countries [1]. When MM is diagnosed early it can be successfully removed by surgical resection, and about 80% of cases are dealt with in this way [2]. However metastatic MM has a very poor prognosis, with a median survival rate of 6 month and a 5-year survival rate of less than 5% [3].

Neuroblastoma is the most common extracranial solid tumor of childhood, and accounts for one of every eight pediatric cancer deaths [4]. The tumor derives from the developing sympathetic nervous system and most primary tumors occur within the abdomen, with at least 50% arising from the adrenal glands [5]. The main feature of neuroblastoma is its remarkable biological heterogeneity, which becomes apparent in the broad variety of the clinical courses of the disease [6]. Besides, at least 40% of all children with neuroblastoma are designated as high-risk patients, meaning that this disease remains a major problem in pediatric oncology.

Both these tumors are refractory to conventional chemotherapy and/or radiation treatment actually in use, hence search for novel therapies is warranted and new therapeutic approaches are needed. C*urcumin *(diferuloylmethane) is the main product extracted from the rhizome of *Curcuma Longa, *a tropical plant native to South and Southeast Asia. It appears as a yellow powder and it is routinely used in the cuisine of the Indian subcontinent as a major component of curry spice. Described in the ancient test of Ayurveda and traditional Chinese medicine for thousands of years, *curcumin *has been used for the treatment of different inflammatory diseases [7]. As a medicine, *curcumin *exhibits remarkable anti-oxidant, anti-inflammatory and anti-cancer activities [8]. Chemopreventive and growth inhibitory activities of *curcumin *against many tumor cell lines, including drug-resistant ones, have been reported [9]. Taking into account the complexity and involvement of multiple signaling pathways in cancer growth and progression, a drug such as *curcumin*, which can interact with multiple target molecules, would be more efficacious than the current mono-targeted anticancer drugs [10]. Indeed, *curcumin *targets several steps in the biochemical pathways leading to cancer (see [9] for a review). It suppresses the expression of cyclin D1, which is deregulated in several types of tumor, and it also induces apoptosis in tumor cells by activating caspase-8, which leads to cleavage of Bid, thus resulting in sequential release of mitochondrial cytochrome *c *and activation of caspase-9 and caspase-3, cleavage of poly ADP ribose polymerase (PARP) and apoptosis of tumor cells. Moreover *curcumin *suppresses the activation of several transcription factors that are implicated in carcinogenesis: it suppresses the activation of nuclear factor kappa B (NFkB), activator protein 1 (AP-1), and at least two of the signal transducer and activator of transcription proteins (STAT3, STAT5). *Curcumin *also modulates expression of genes involved in cell proliferation, cell invasion, metastasis, angiogenesis, and resistance to chemotherapy [11] and it shows a potent chemopreventive activity against a wide variety of tumors. Recently, *curcumin *has been reported to exert a good antiproliferative activity on melanoma cells by inducing apoptosis [12]. In several types of human melanoma cells, *curcumin *induces apoptosis through the Fas receptor/caspase-8 pathway independent of p53 and suppresses the antiapoptotic gene XIAP [13]. Overall, several pilot clinical trials using *curcumin *against various tumors have been reported; however, its concentration levels in serum and tissues have been demonstrated to remain very low due to its poor bioavailability and high instability under physiological conditions [9].

A number of *curcumin *related compounds have been synthesized by our group and tested *in vitro *in order to select new antitumor agents displaying stronger and selective growth inhibition activity on melanoma cells (unpublished data). It has been previously reported that hydroxylated biphenyl structures could be effective as cytotoxic agents in MM and NB cells, with apoptotic inducing capability [14]. On this basis we evaluated the possibility to design a molecule that, while keeping or improving the biological properties of *curcumin*, could exploit the activity of hydroxylated biphenyl compounds, which are generally more bioavailable. In this paper, we describe the synthesis of the new *curcumin*-related biphenyl compound D6, an α,β-unsaturated ketone, and test its anticancer properties. Antiproliferative and proapoptotic activity of D6 has been assessed by *in vitro *experimental procedures on MM and NB cell lines showing D6 more effective than *curcumin *in inhibiting tumor cells growth and inducing apoptosis by involving the intrinsic pathway. *In vivo *assays on both MM and NB mouse models have also been carried out, confirming the D6 anticancer potentiality.

## Methods

### Chemicals

**Figure 1:**
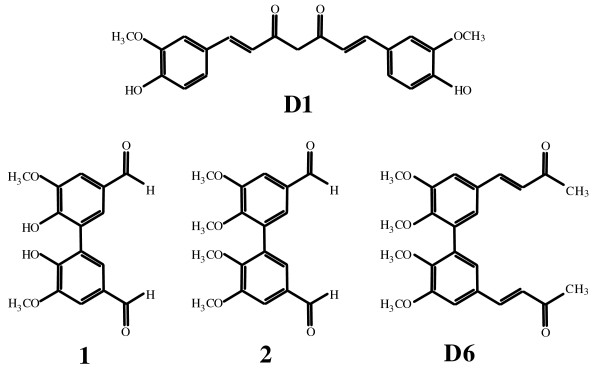
**Chemical structures**. *Curcumin *D1 and related biphenyl D6 with its precursors (1) and (2).

*Curcumin *(**D1**, Figure 1) was purchased from Alfa Aesar (GmbH & Co KG, Karlsruche, Germany), with a purity of 95%, dissolved in dimethyl sulfoxide (DMSO) at a final concentration of 100 mM and stored at -20°C until used. For *in vitro *experiments, D1 was diluted in complete medium to contain <0.1% DMSO, immediately before use. For *in vivo *experiments, D1 was diluted in a sterile 0.9% (w/v) NaCl solution containing 1.65 mg/ml bovine serum albumin (BSA, Sigma, St. Louis, MO, USA) and 3.6% DMSO (v/v), as reported with slight modifications [15,16]. D1 dilutions were freshly prepared immediately before injection and protected from light.

Pan-caspase inhibitor (z-VAD-FMK) was purchased from Sigma.

### Synthesis of D6

The new *curcumin*-related biphenyl compound, the α,β-unsaturated ketone **D6 **was synthesized as follow (Figure 1):

Preparation of *2,2',3,3'-tetramethoxy-5,5'-diformyl-1,1'-biphenyl *(**2**).

CH_3_I (1.41 g, 9.93 mM) was added, dropwise at RT under N_2_, to a solution of *2,2'-dimethoxy-3,3'-dihydroxy-5,5'-diformyl-1,1'-biphenyl *(**1**) [17] (1.0 g, 3.31 mM) and K_2_CO_3 _(1.37 g, 9.93 mM) in 20 ml of dry acetone. The solution was stirred at reflux for 12 hours and washed with water (100 ml). The organic phase was extracted with ether (2 × 100 ml) and dried over Na_2_SO_4 _to obtain a yellow solid. The crude material was purified by flash chromatography using a 6:4 mixture of petroleum:acetone as eluent to give (**2**) as a white solid. (0.87 g, 80%): mp 130-131°C; ^1^H NMR δ 3.76 (s, 6H), 3.97 (s, 6H), 7.38 (d, *J *= 2.0 Hz, Ar, 2H), 7.49 (d, *J *= 2.0 Hz, Ar, 2H), 9.90 (s, 2H); ^13^C NMR δ 56.07, 60.99, 110.17, 127.44, 131.57, 131.87, 152.11, 153.15, 190.69; Anal. Calcd for C_18_H_18_O_6_: C, 65.45; H, 5.49; Found: C, 65.55; H, 5.38.

Preparation of *(3E,3'E)-4,4'-(5,5',6,6'-tetramethoxy-[1,1'-biphenyl]-3,3'-diyl)bis(but-3-en-2-one) *(**D6**).

A 1 N solution of NaOH (1.6 ml) was added, dropwise, to a solution of *2,2',3,3'-tetramethoxy-5,5'-diformyl-1,1'-biphenyl *(**2**) (1.65 g, 0.5 mM) in dry acetone (10 ml). The solution was stirred at RT for 12 hours. The solvent was evaporated. 10% HCl was added (100 ml), the organic phase was extracted with dichloromethane (2 × 100 ml) and dried over Na_2_SO_4 _to obtain a yellow solid. The crude material was purified by flash chromatography using a 6:4 mixture of petroleum: acetone as eluent to give (**D6**) as a white solid. (1.07 g, 52%): mp 166-167°C; ^1^H NMR δ 2.35 (s, 6H), 3.69 (s, 6H), 3.95 (s, 6H), 6.63 (d, *J *= 16.4 Hz, 2H), 7.05 (d, *J *= 2.0 Hz, Ar, 2H), 7.11 (d, *J *= 2.0 Hz, Ar, 2H), 7.45 (d, *J *= 16.4 Hz, 2H); ^13^C NMR δ 27.46, 55.91, 60.85, 110.80, 124.10, 126.47, 129.82, 132.32, 143.04, 149.05, 153.02, 198.22; Anal. Calcd for C_24_H_26_O_6_: C, 70.23; H, 6.38; Found: C, 69.89; H, 6.48.

### Cell lines and culture conditions

Malignant melanoma (MM) cell lines [LB24Dagi (LB24), CN-MelA (CN), GR-Mel (GR), WM266-4 (WM), 13443, and M14] were a gift from Dr. D. Castiglia at Istituto Dermopatico dell'Immacolata, Department of Molecular and Cellular Biology, in Rome, as reported [14]. The following human neuroblastoma (NB) cell lines were used: GI-LI-N, HTLA-230, SH-SY5Y, LAN5, SK-NBE2c, and IMR-32 [18,19]. Normal human fibroblasts BJ (CRL-2522) were purchased from the American Type Culture Collection (ATCC) and served as controls. All cells were grown either in Dulbecco's minimal essential medium (DMEM, Sigma) or in RPMI medium (Invitrogen, Carlsbad, CA, USA), as described [14,18,19].

### Cell proliferation assay

All MM (3-5 × 10^3 ^per well) and NB (8-12 × 10^3 ^per well) cell lines were plated in 96-well plates in complete medium and treated in quadruplicate with different concentrations of either D1/D6 (0-25 μM) or 0.1% DMSO (control) for 72 hours. The percentage of MM cell proliferation was estimated on day 5 by the colorimetric assay of Kueng *et al. *[20] modified as previously reported [21]. Neuroblastoma cells were incubated overnight with 0.5 μCi (0.0185 MBq) ^3^H-thymidine (Amersham Bioscience, Little Chalfont, UK) and processed for liquid scintillation counting (Packard Instruments Company, Downers Grove, IL, USA), as described [19].

To assess the effect of duration of drug exposure on cell proliferation, we also performed drug washout experiments, according to the method described by Keshelava *et al. *[22]. Briefly, the D1*/*D6-containing medium was removed from the cultures after 6 or 12 hours by washing the cells twice with complete medium. The cells were subsequently covered with fresh drug-free complete medium and incubated for a total incubation time of 72 hours. Cell proliferation was evaluated by either measuring DNA synthesis as a function of ^3^H-thymidine uptake or by the colorimetric assay, as above [19,21].

### Clonogenic cell survival assay

MM (LB24) and NB (GI-LI-N) cells were suspended at concentrations of 3 × 10^5 ^cells/ml, treated with either D1 or D6 (0, 2.5, 5, 10, or 20 μM) in culture medium for 4 hours and then plated (in triplicate) at 50 (LB24) and 300 (GI-LI-N) cells per well into 12-well plates with fresh drug-free medium. Cells were incubated for an additional 14 days, then the colonies in each well were stained, counted and photographed [18].

### Apoptosis assays

#### Terminal deoxynucleotidyl transferase-mediated dUTP nick end labeling (TUNEL) assay

LB24 melanoma cells and BJ fibroblasts were plated in 8 well-chamber slides (5 × 10^4 ^and 5 × 10^5^/well, respectively), cultured for 24 hours, and then treated with either D1 or D6 (10 μM). After further 24 hours, DNA cleavage was assessed by enzymatic end-labeling of DNA strand breaks using a commercial kit (In Situ Cell Death Detection Kit, Roche, Penzberg, Germany), according to the manufacturer's instructions and as previously reported [14].

#### Phosphatidylserine detection

For detection of phosphatidylserine exposure, cultured LB24 cells (1 × 10^6^/25 cm^2 ^plastic culture flask) and GI-LI-N cells (2 × 10^6^/25 cm^2 ^plastic culture flask) were treated with either D1 (10 μM) or D6 (5 μM and 10 μM), collected, washed and processed using a human Annexin-V FITC kit (Bender MedSystems, Vienna, Austria), according to manufacturer's instructions and examined by two-color flow cytometry using a FACScan device [18,14].

#### Caspase 3/7 activation

Detection of caspase 3/7 cleavage activity was done using the Apo-ONE^R ^Homogeneous Caspase 3/7 Assay (Promega, Madison, WI, USA). GI-LI-N, SH-SY5Y, IMR-32, LB24 and M14 cell lines were seeded in 96-well plates (10-20 × 10^3^/well). Cells were then treated with D1 or D6 at a concentration of 10 μM and 5 μM, respectively, for 24 hours. In some experiments, cells were treated for 1 hour with 100 μM z-VAD-FMK caspase inhibitor before D1 and D6 administration. At the end of treatment, 100 μl of the Apo-ONE Caspase 3-7 Reagent was added to each well, according to manufacturer's instructions. Cells were then incubated at room temperature in a plate shaker (400 rpm) for 3 hours. Fluorescence was detected (485 nm excitation wavelength and 535 emission wavelength) using a fluorescence plate reader (SPECTRAFluor Plus, TECAN Austria, GmbH, Grodig/Salzburg, Austria). The amount of fluorescent product generated is proportional to the amount of caspase 3/7 cleavage activity present in the sample.

#### Mitochondrial membrane potential assay

The mitochondrial permeability transition event was detected by MitoPT kit (Immunochemistry Technologies, LLC, Bloomington, MN, USA) according to the manufacturer's instructions. Briefly, IMR-32 and LB24 cell lines were plated in 6-well flat-bottomed plates (0.7 × 10^6^/well and 0.4 × 10^6^/well, respectively). The cells were then treated with both D1 and D6 (2.5-10 μM and 5-10 μM, respectively) for 18 hours, harvested, and processed as previously described [18,19]. The analysis was performed using a FACS Calibur (Beckton Dickinson, Franklin Lakes, NJ, USA). As the mitochondrial membrane potential collapsed (apoptosis), the amount of red fluorescence decreased.

### Western blot analysis

Total cell lysates were prepared and analyzed by western blot analysis as described earlier [18,19]. Briefly, both MM and NB cell lines were untreated or treated with either D1 (10 μM) or D6 (5 μM) for 18, 24 or 48 hours and then lysed with Cell Extraction Buffer (BioSource International, Camarillo, CA, USA) plus protease inhibitor cocktail (Sigma). To obtain subcellular fractions, the cells were processed with Qproteome Cell Compartment kit, according to the manufacturer's instructions (Qiagen, Crawley, UK), and resuspended with the above-mentioned lysis buffer. Protein lysates (50 μg per lane) were resolved on sodium dodecyl sulphate (SDS) 10 to 14% polyacrylamide gels and transferred to nitro-cellulose membranes; the membranes were then incubated with mouse monoclonal antibodies against procaspases 3, 9 (Cell Signaling Technology, Danvers, MA, USA) and NFkB (BD), or rabbit monoclonal antibody against PARP (Abcam, Cambridge, UK) or polyclonal antibody against cytochrome c (Cell Signaling Technology). Peroxidase-conjugated goat anti-mouse and anti-rabbit antibodies were used as secondary antibodies (Upstate, Lake Placid, NY, USA and Santa Cruz Biotechnology, Santa Cruz, CA, USA, respectively). Immune complexes were visualized with the use of an ECL Advance Western Blotting Detection kit (Amersham Bioscience), according to the manufacturer's instructions, and normalized to internal controls [a rabbit antibody against GAPDH (glyceraldehyde-3-phosphate dehydrogenase) (Sigma) and a mouse antibody against Lamin A/C (BD)].

### Animal models

All animals were purchased from Harlan Laboratories (Harlan Italy, S.Pietro al Natisone, Italy) and housed under specific pathogen-free conditions. All experiments involving animals have been reviewed and approved by the licensing and ethical committee of the National Cancer Research Institute, Genoa, Italy and by Italian Ministry of Health. All the *in vivo *experiments were performed using 5-week-old female athymic (nude-*nu*), 5 to 8 mice per group and repeated at least twice with similar results.

For the melanoma animal model, 1.5 × 10^6 ^LB24 cells were injected subcutaneously (*s.c.*) in the mid-dorsal of the mice, as previously reported [23]. Tumors were allowed to grow for 5 days, reaching a size of about 10 mm^3^, before intravenous (*i.v.*) treatment into tail vein.

For the neuroblastoma animal model, 1.5 × 10^6 ^GI-LI-N cells were orthotopically injected in the capsule of the left adrenal gland of mice, as described previously [24,19]. In another set of experiments, mice were orthotopically injected with 1.5 × 10^6 ^luciferase-transfected GI-LI-N cells to monitor *in vivo *orthotopic tumor growth over time by bioluminescence imaging (BLI) [25].

### *In vivo *therapeutic studies

In order to administer D1 and D6 by intravenous route (for a rapid and efficacious drug distribution), we have pre-dissolved curcuminoids in DMSO at 10 mM and then diluted in a saline solution containing BSA, as previously suggested by others (15, 16) and by us (19) for water-insoluble compounds. In the melanoma animal model, after tumor growing as above, mice were randomly assigned to three groups (8 mice/group) and treated with D1, D6 or with saline solution (control mice). Either D1 or D6 were *i.v. *injected at 17.5 mg/Kg twice a week for a total of 7 times. Moreover, at both 2 hours post the first injection and one day after the last one, four mice of each group of treatment were subjected to blood sample to test acute and chronic liver and renal toxicity using an automatic analyzer Kuadro Liquid Vet (BPC BioSed, Rome, Italy). Tumors were measured twice weekly with calipers and volume were calculated by the formula 4/3 π axbxc where a, b and c represented the values of all symmetric axes that constitute the ellipsoid tumor mass. At the end of the treatment, mice were killed by cervical dislocation after being anesthetized with xylazine (Xilor 2%; Bio98 Srl, Milan, Italy), and their tumors were measured with caliper and then weighted.

For neuroblastoma animal model, tumors were allowed to grow for 14 days, and then mice, randomly assigned to three groups (8 or 5 mice/group), were treated with the same schedule used for the above melanoma animal model.

In all experiments, body weight and general physical status of the animals were recorded daily until they were judged to be in discomfort by animal caretakers. Specifically, once showing signs of poor health (i.e., abdominal dilatation, dehydration, paraplegia, severe weight loss) mice were euthanized, following anesthesia with xylazine and the day of euthanasia was recorded as the day of death.

### Statistical analysis

All *in vitro *data derive from at least three independent experiments and results are expressed as mean values with 95% confidence intervals. The statistical significance of differential findings between experimental and control groups was determined by ANOVA with the Tukey's multiple comparison test in Graph-Pad Prism 3.0 software (Graph-Pad Software, Inc, San Diego, CA, USA). These findings were considered significant if two-tailed *P *values were <0.05. Survival curves have been constructed with the Kaplan Meier method. All the *in vivo *experiments were performed at least twice with similar results. A *P *value less than 0.05 has been considered as statistically significant by the use of the Peto's log-rank test in Graph-Pad Prism 3.0 software.

## Results

### Effects of D1 and D6 on proliferation, clonal growth and viability of melanoma and neuroblastoma cell lines

**Figure 2:**
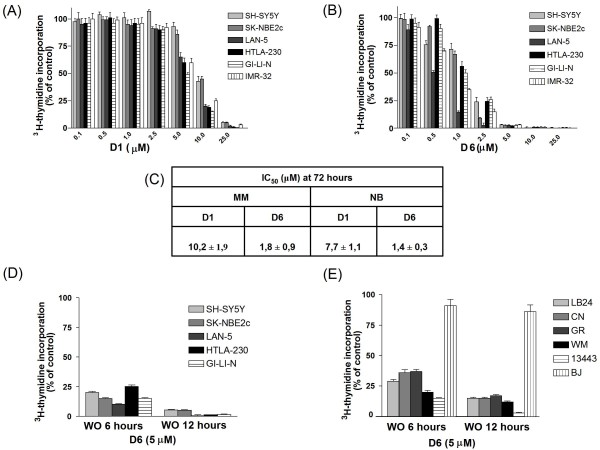
**Effects of D1 and D6 on the growth of human neuroblastoma and melanoma cell lines**. (**A**), (**B**): Six NB cell lines were cultured in the presence of various concentrations (0-25 μM) of D1 (A) and D6 (B) or 0.1% DMSO (control cells) for 72 hours. Cell proliferation was measured by ^3^H-thymidine incorporation. Results, derived from three different experiments, are expressed as mean percentage of ^3^H-thymidine incorporation from quadruplicate wells as compared to that of control cells. Error bars represent 95% confidence intervals. (**C**) IC_50_ values obtained on the six NB cell lines and the six MM cell lines reported in the M&M section. (**D**) and (**E**): drug washout assays to assess the impact of D6 time exposure on cell proliferation in NB cells (D), MM cells and BJ fibroblasts (E). Cells were incubated with D6 for the indicated times and concentration and then washed and cultured in drug-free medium up to 72 hours. Cell proliferation was assessed by ^3^H-thymidine incorporation. Results are expressed as described above.

Cell proliferation assays were performed to assess the cytotoxic activity of the novel biphenyl, *curcumin*-related compound D6 (see Figure 1) in comparison with that of *curcumin *D1. Figure 2 shows the dose-dependent cell growth inhibition of 6 NB cell lines treated with either D1 (panel A) or D6 (panel B), clearly evidencing the stronger efficacy of D6 at very low doses (1-2.5 μM) after three days of treatment. MM cells growth inhibition gave similar results and a summary of all the IC_50 _values obtained at 72 hours for MM and NB cell lines for both compounds is reported in figure 2, panel C. Considering the IC_50 _mean values ± s.d. obtained for all NB and MM cell lines tested we can note that D6 is about 5-10 fold more efficient than D1 in both cases.

To assess the impact of D6 exposure time on cell proliferation, washout experiments were performed. These experiments simulated the *in vivo *activity of D6, testing the minimum time of response of the tumor cells to the drug. D6 action was very powerful and rapid in arresting NB and MM cells growth: a single 6 hours treatment was sufficient to inhibit tumor cells proliferation up to 75-90%, having a small effect (10-20%) on BJ normal fibroblasts (Figure 2, panels D, E). Moreover cell proliferation capability was not restored when cells were grown in the absence of the drug up to 72 hours, showing that the antiproliferative effect of D6 was irreversible. Washout experiments performed using D1 instead, did not show any appreciable activity on cell proliferation for both type of tumor cells (Additional file 1 Figure S1).

**Figure 3:**
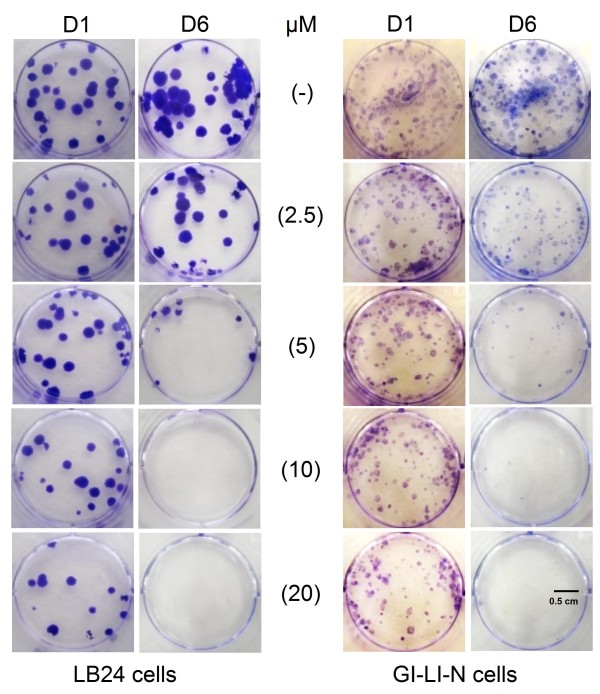
**Effects of D1 and D6 on the clonogenic growth of melanoma (LB24) and neuroblastoma (GI-LI-N) cell lines**. Cells have been suspended in drug-containing medium at different concentrations (0-20 μM) of D1 and D6 for 4 hours and then washed, seeded with drug-free medium in a 12 well-plate and let grow up to 14 days. Cell colonies were stained with crystal violet, counted and photographed as described in M& M.

To measure long-term effects of D1 and D6 on permanent cell growth arrest and cell death we performed clonogenic survival assays. Clonal growth of both LB24 melanoma cells and GI-LI-N neuroblastoma cells was inhibited by D6 in a dose dependent manner as shown in figure 3. Conversely, D1 was not able to abolish colony formation under the same conditions, but partly inhibited clonogenic growth only at the highest concentration used (20 μM).

### D1 and D6 induce apoptosis in both melanoma and neuroblastoma cells

**Figure 4:**
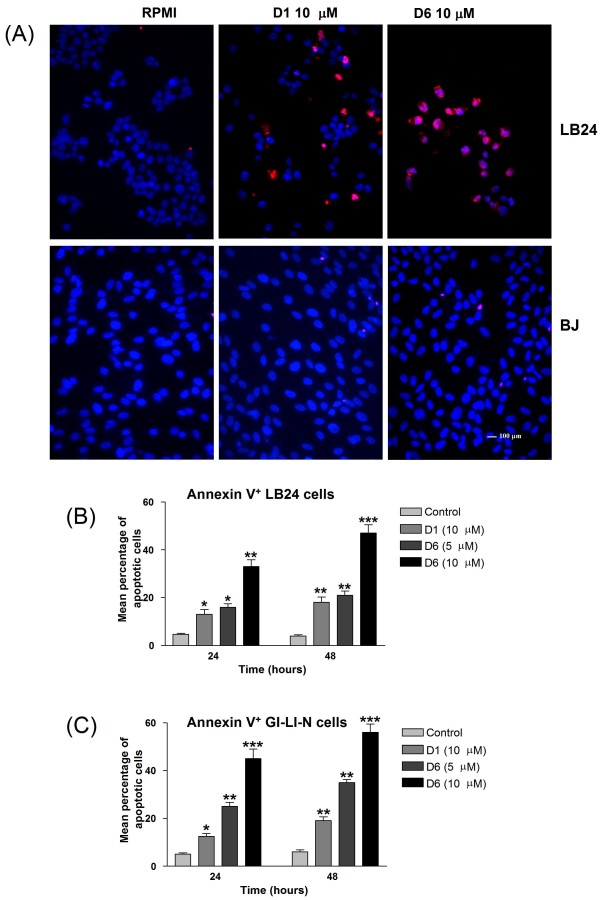
**Effects of D1 and D6 on apoptosis in melanoma and neuroblastoma cells**. (**A**) Melanoma cells (LB24) and normal fibroblasts (BJ) were incubated with solvent (control), or with 10 μM D1 and D6 for 24 hours, then stained by TUNEL assay. Apoptotic cells are visualized by red staining. Cell nuclei (blue) were stained with DAPI (bar, 100 μm). (**B**) Melanoma cells (LB24) and (**C**) Neuroblastoma cells (GI-LI-N) were incubated with 10 μM D1 and with 5 and 10 μM D6 for 24 and 48 hours. Apoptosis induction was determined as the percentage of Annexin-V positive cells. Columns: means; bars: SD. *, *P *< 0.05; **, *P *< 0.01; ***, *P *< 0.001 *vs *control levels.

To determine whether the observed D1- and D6-induced reduction in viability of both melanoma and neuroblastoma cell lines occurred via activation of apoptosis, we used TUNEL staining to highlight DNA fragmentation and Annexin-V assay, in which Annexin-V binds to externalized phosphatidylserine on the surface of apoptotic cells. TUNEL assay in figure 4, panel A shows a higher proportion of apoptotic melanoma cells in the D6 treated sample compared to the D1 treated one, while human BJ fibroblast cells appear not to be affected by these treatments. Furthermore, when LB24 melanoma cells were exposed for 24 and 48 hours to 5 and 10 μM of both D1 and D6, a significant, time- and dose-dependent, increase in the percentage of Annexin-V-positive cells was observed, particularly following D6 treatment (Figure 4, panel B). Similar results were obtained with GI-LI-N neuroblastoma cells (Figure 4, panel C). (*, *P *< 0.05; **, *P *< 0.01; ***, *P *< 0.001 *vs *untreated cells).

**Figure 5:**
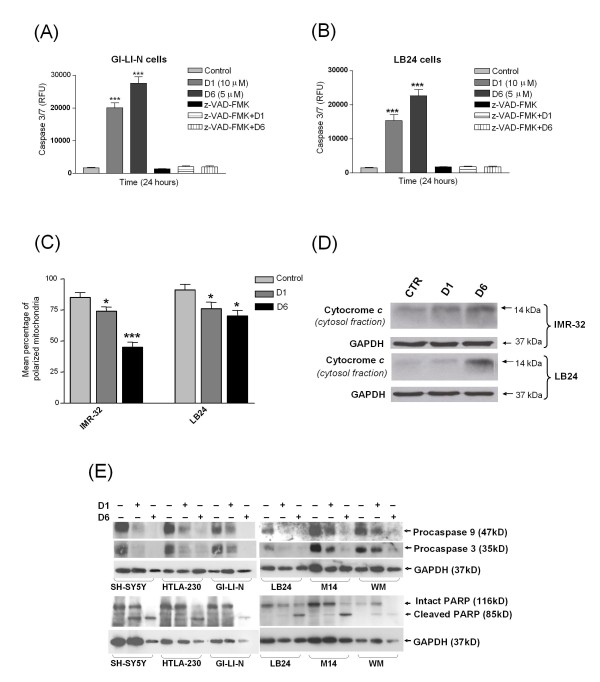
**D6 and D1 triggered-apoptosis is caspase-dependent and involves the apoptotic intrinsic pathway**. (**A**) and (**B**): Caspase 3/7 activation was assessed on GI-LI-N cells (**A**) and LB24 cells (**B**), untreated (control), or treated with D1 (10 μM) and D6 (5 μM) for 24 hours. Cells received an 1 hour pre-treatment with a pan-caspases inhibitor (z-VAD-FMK). RFU: Relative Fluorescence Unit. ***, *P *< 0.001 *vs *control. (**C**) To assess the effects of drugs on mitochondrial membrane permeability, neuroblastoma IMR-32 and melanoma LB24 cells were untreated (control) or treated with D1 and D6, as above, for 18 hours, harvested, and analyzed for changes in mitochondrial membrane potential. Results are expressed as the mean percentage of cells with polarized mitochondria from four independent experiments; error bars, 95% CIs. *, P < 0.05; ***, P < 0.001 *vs *control. (**D**) IMR-32 and LB24 cells were treated for 24 hours as above and fractionated cellular lysates were analyzed by western blot with a specific primary antibody for cytochrome *c*. GAPDH was used as loading control. (**E**) SH-SY5Y, HTLA-230, GI-LI-N neuroblastoma cell lines and LB24, M14, WM melanoma cell lines were treated as in panel D for 48 hours and total cellular lysates were analyzed by western blot with specific primary antibodies for procaspase 9, procaspase 3 and poly(ADP)ribose polymerase (PARP). As for (D), blots are representative of three independent experiments and data were normalized against GAPDH.

Activation of aspartate specific cysteine proteases or caspases may be involved in induction of apoptosis [26]. Caspase 3/7 assays showed that both D1 and D6 (at 10 μM and 5 μM, respectively) significantly induced the cleavage of caspases 3 and 7 in GI-LI-N neuroblastoma cells and LB24 melanoma cells (Figure 5, panels A and B, respectively). In both cases D1 and D6 *vs *control showed a very significative value ***, *P *< 0.001 and the effect of D6 was stronger when compared to D1 for both cell lines. A pre-incubation with the pan-caspase inhibitor z-VAD-FMK completely abolished the D1 and D6-induced caspases 3 and 7 cleavage activity, meaning that caspases are involved in this cell death. Since activation of caspases 3 and 7 has been reported to also belong to the intrinsic apoptotic signaling pathway [27,28], we decided to investigate mitochondria-related events, mitochondrial membrane damage and release of the cytochrome *c *into the cytosol, which is putatively involved in the curcuminoids-triggered cell death. The percentage of both IMR-32 and LB24 cells with polarized mitochondria decreased after 18 hours treatment with the above mentioned doses of D1 and D6: this reduction was statistically significant and highlighted, also in this context, the major sensitivity of neuroblastoma cells to D6 (Figure 5, panel C: IMR-32: D1 *vs *control: *, *P *< 0.05 and D6 *vs *control: ***, *P *< 0.001; LB24: both D1 and D6 *vs *control: *, *P *< 0.05). The resulting release of cytochrome *c *from mitochondria appeared more consistent after D6 treatment in both IMR-32 and LB24 cell lines (Figure 5, panel D). Consistently, this event led to caspase 9 activation in both NB and MM cells, indicating that, together with the disruption of mitochondrial membrane potential, the intrinsic apoptotic pathway was involved in this gene activation cascade. Moreover, the level of pro-caspase 3 was also decreased in response to D6 treatment and this event culminated in the cleavage of poly(ADP)ribose polymerase (PARP), confirming the occurring of programmed cell death (Figure 5, panel E).

### *In vivo *anti-tumor activity of D1 and D6 against human neuroectoderma-derived xenografts

We used two previously described animal models, a subcute tumor model for melanoma [23] and an orthotopic xenograft model for neuroblastoma [19] to evaluate whether D1 and D6 would inhibit neuroectoderma-derived cell growth *in vivo*.

In cancer related research, curcuminoids have been used in cell culture systems, animal models and clinical trials [9,29,30]. Unfortunately, the study of curcuminoids is severely limited by their extremely low bioavailability following oral administration. Moreover, only predissolving curcuminoids in DMSO render them in a physical form, that is proper for solubilization in aqueous media containing serum or preferentially BSA [16]. Then, in a set of preliminary experiments, we have injected D1 and D6 in the tail vein and we have been able to obtain good animal responses to a restrict range of administered doses (15-25 mg/Kg), in terms of good health (i.e., no abdominal dilatation, dehydration, paraplegia, severe weight loss), together with slightly variations in both liver and renal enzymatic values, only at the higher doses throughout the experiments. Therefore, after both subcutaneous injection of LB24 melanoma cells and orthotopic implantation of GI-LI-N neuroblastoma cells, we treated mice with 17,5 mg/Kg of D1 and D6 by *i.v. *injection twice a week, for 7 times.

**Figure 6:**
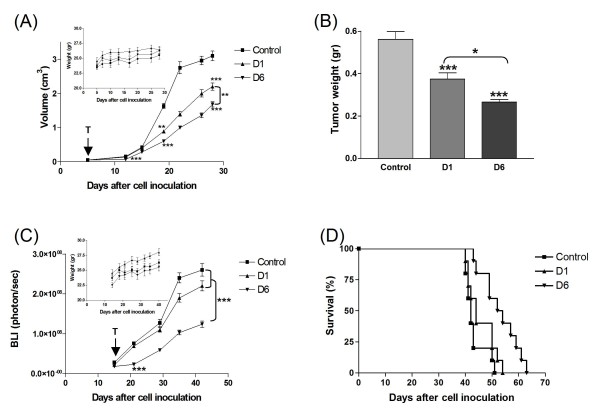
***In vivo *anti-tumor activity of D1 and D6**. (**A**), (**B**): in the melanoma animal model, nude mice (n = 8 mice/group) received *i.v. *17.5 mg/kg of D1 and D6 or saline solution (control mice) on day 5 after *s.c. *injection of 1.5 × 10^6 ^LB24 cells, twice a week, for 7 times. (**A**) Tumor volumes were measured at different times points after cell inoculation. *P *values were calculated using Peto's log-rank test (**, *P *< 0.01; ***, *P *< 0.001). **Inset**: absolute mean body weight, in grams, after the beginning of the treatment. (**B**) Two days after the end of the treatment, mice were sacrificed and tumors were excised and weighted. Bars depict the mean values and error bars represent 95% confidence intervals. *P *values were calculated using ANOVA with Tukey's multiple comparison test (*, *P *< 0.05; ***, *P *< 0.001). (**C**): Nude mice were orthotopically injected with 1.5 × 10^6 ^luciferase-transfected GI-LI-N neuroblastoma cells and tumors were allowed to grow for 14 days, then mice, randomly assigned to three groups (5 mice/group), were treated with the same schedule used for the above melanoma animal model. Orthotopic tumor growth was monitored over time by bioluminescence imaging (BLI). *P *values were calculated using Peto's log-rank test (***, *P *< 0.001). **Inset**: absolute mean body weight, in grams, after the beginning of the treatment. (**D**) Therapeutic effects on survival were evaluated on nude mice (n = 8 mice/group) orthotopically injected with 1.5 × 10^6 ^GI-LI-N cells in the left adrenal gland and treated as above. Survival of mice was monitored daily. *P = 0.0007 *for D6 over control and *P = 0.0169 *for D6 over D1.

In melanoma-bearing mice (Figure 6, panel A), both D1 and D6 induced a significant inhibition of tumor growth when compared to that of untreated mice. Noteworthy, the D6 inhibitory effect was already evident at early days of treatment, and it was more statistically efficacious than D1 (D6 *vs *D1, *P < 0.01*). The pronounced delay in tumor growth, especially by D6, was translated into tumor weight values, which have been obtained at the end of the treatment (Figure 6, panel B: both D1 and D6 *vs *control, ***, *P *< 0.001; D6 *vs *D1, *, *P *< 0.05).

Even in the neuroblastoma animal model, D6 induced a statistically significant more pronounced tumor growth reduction than D1 evaluated by means of BLI (Figure 6, panel C: D6 *vs *both D1 and control, *P < 0.001*). More importantly, the survival rate was significantly increased only by the D6 treatment (Figure 6, panel D: D6 *vs *control, *P = 0.0007; *D6 *vs *D1, *P = 0.0169*).

In the two experimental animal models, the mean body weight of both D1 and D6-treated mice was not significantly lower than that of saline-injected (control) mice, indicating that the molecules had minimal side effects (Insets, Figure 6, panels A and C). Besides, this good systemic tolerability was also confirmed by the trend of both liver and renal enzymatic values throughout the experiments (Additional file 2 Table S1).

## Discussion

Despite the current advances in anticancer therapies, the clinical prognosis of both malignant melanoma and aggressive neuroblastoma remain fatal. For this reason search of effective novel drugs, with low systemic toxicity, is a main field in oncology research.

*Curcumin*, which is a natural derived compound, considered pharmacologically safe even at high doses [31], has been shown to prevent tumor formation and progression in many cancer types as well as to exert growth inhibitory effects on several neoplastic cells [10]. In this study, we demonstrated that a newly-synthesized *curcumin*-related biphenyl compound, the α,β-unsaturated ketone D6, acts as a very promising anticancer molecule, being more effective than *curcumin *in inhibiting neuroectodermal derived cancer cells growth by inducing apoptosis. In particular, D6 caused proliferation arrest in cultured NB and MM cells at low concentrations (1-5 μM), whereas *curcumin *reached the same effects at concentrations 5-10 fold higher. Moreover D6 treatments had no effect on normal fibroblasts at the same concentrations suggesting that this compound shows preferential cytotoxic activity on cancer cells. *In vitro *response to a given drug could be influenced by cell culture conditions: usually, cells grown in monolayer are more sensitive to cytotoxic agents than cells grown in colonies because of the larger surface they expose to the drug, compared to the limited drug penetration in the colonies [32]. Moreover *in vivo *growth rate of solid tumors is typically lower than that of *in vitro *monolayer cultures. Our results showed that D6 is similarly efficient in suppressing *in vitro *proliferation of NB and MM cells in both monolayer and colony cell culture conditions. This finding suggests that D6 could probably exploit its *in vivo *anticancer activity without being influenced by the low fraction of proliferating cells in solid tumors, which is a limit of many of the conventional cell cycle-dependent chemotherapeutic agents [33].

Several studies indicated *curcumin *as a proapoptotic agent against different types of cancer cells (reviewed in [9]); therefore, we investigated whether D6-driven proliferation arrest was due to induction of apoptosis. In our experiments, both NB and MM cells presented apoptosis following D6 treatments. Moreover, a more consistent portion of apoptotic MM cells was observed in cells treated with D6 compared to those treated with *curcumin*. Interestingly, no evidence of apoptosis was observed in normal human BJ fibroblasts grown in the same conditions, again suggesting the preferential antiproliferative activity of D6 on cancer cells.

Induction of apoptosis may also involve activation of aspartate specific cysteine proteases or caspases. Our results show that activation of both caspase 3 and caspase 7 occurred after treatment of NB and MM cells with D6, again being more efficient than the treatment with D1. One of the hallmark of caspase activation is polyadenosine-5'-diphosphate-ribose-polymerase (PARP) cleavage, where the native 116 kD polypeptide is cleaved to a smaller 85 kD fragment. Usually, PARP inactivation is dependent on caspase 3, which has a central role in the apoptosis process collecting signals coming from both intrinsic and extrinsic pathways. Induction of apoptosis by *curcumin *has been reported to occur through both mitochondrial intrinsic pathway [34] and death receptor mediated extrinsic pathway [13]. Cleavage of procaspase 9 in the D6 treated cells suggested the involvement of mitochondrial pathway in the mechanism of action of this compound; indeed, this hypothesis was validated by the observation of mitochondrial membrane depolarization. Noteworthy, all the apoptosis hallmarks were found significantly higher in D6-treated than *curcumin*/D1-treated cells, strongly suggesting that D6 may act as a more effective proapoptotic agent.

Considering our *in vivo *data, both NB and MM xenografts showed a good response to the D6 treatments: tumor mass growth was significantly reduced compared to control mice, especially after the D6 administration. Importantly, the survival time increased in a statistically significant way among D6 treated mice, while no evident side effects were observed. On this regard, mean body weight of treated mice was not affected by either D1 or D6 administration and both liver and renal functionality showed to be normal (see Additional file 2 Table S1). These findings confirmed that this class of compounds, such as *curcumin*, has good tolerability and an interesting anticancer proapoptotic activity.

*Curcumin *has been reported to act as anticancer agent by suppressing NFkB signaling (reviewed in [35] and [9]). Constitutively active form of NFkB has been reported in several human cancers [36] and activated NFkB suppresses apoptosis in a wide variety of cancer cells [37]. When we investigated the possible involvement of NFkB signaling suppression in D6 antiproliferative activity we found out that our cell lines did not show constitutive activation of this transcription factor so that probably this pathway is not involved in the transformation processes of these cells (see Additional file 3 Figure S2). The *curcumin *and D6 antiproliferative and proapoptotic action on our MM and NB cells must be therefore due to different mechanisms. Nevertheless, when NFkB activation and nuclear translocation was induced in NB cells by TNFα or doxorubicin pretreatments, D1 and, even better, D6 administration to the cells consistently inhibited NFkB activation, indicating the capability of these compounds to interfere with such molecular activation of transcription pathway. The last evidence points out the potentiality of D6 to be used in combination with chemotherapeutic agents which stimulate the activation of gene transcription through NFkB pathway, such as doxorubicin [38].

## Conclusions

In summary, we have demonstrated that hydroxylated biphenyls structurally related to *curcumin *are a class of molecules worth to be studied for its promising antiproliferative and proapoptotic features. Moreover, although a large selection of *curcumin *analogues have been published and tested as antitumoral agents [39], no examples of hydroxylated biphenyl *curcumin*-related have been published before. Specifically, D6 activity seems to be effective, rapid, and selective against both NB and MM cells, by causing apoptosis *in vitro *and reduction of tumor mass growth in mice models *in vivo*. Such an antiproliferative activity seems to be achieved using lower concentrations than *curcumin*. As a consequence, D6 could be considered a good candidate to develop new therapeutic strategies against tumors with neuroectodermal origin.

## Competing interests

The authors declare that they have no competing interests.

## Authors' contributions

MPisano carried out melanoma cell cultures, cell proliferation assays, clonogenic cell survival assays, western blot analysis, TUNEL assays and drafted the manuscript. GPagnan carried out neuroblastoma cell cultures, cell proliferation assays, phospatidylserine detection, caspase 3/7 activation assays, mitochondrial membrane potential assay, western blot analysis, *in vivo *therapeutic studies and drafted the manuscript. MAD, DF and GD performed the chemical synthesis and the resolution of the *curcumin*-related biphenyl compound D6. SC and IS participated to melanoma cell cultures, cell proliferation assays, clonogenic cell survival assays, western blot analysis and TUNEL assays. IC participated to neuroblastoma cell cultures, cell proliferation assays, phosphatidylserine detection, caspase 3/7 activation assays, mitochondrial membrane potential assay, western blot analysis, *in vivo *therapeutic studies. LE and MC carried out animal models establishments and animals care. FP participated to animal models establishment and *in vivo *therapeutic studies. GPalmieri contributed to the final drafting and critical revision of the manuscript. MPonzoni performed statistical analysis, CR carried out western blot analysis and together they conceived of the study, participated in its design and coordination and carried out the final drafting of the manuscript. All authors read and approved the final manuscript.

## Supplementary Material

Additional file 1 **Figure S1**. Effects of D1 on melanoma and neuroblastoma cell proliferation analyzed with drug washout experiments Click here for file

Additional file 2 **Table S1**. Liver and renal toxicity values during the *in-vivo *therapeutic studies Click here for file

Additional file 3 **Figure S2**. Effects of D1 and D6 on NFkB expression in neuroblastoma cell line (figure, legend and methods) Click here for file
