# Supplementary material for: Enhanced anti-tumor activity of a new curcumin-related compound against melanoma and neuroblastoma cells
Source: Mol Cancer. 2010 Jun 3;9:137. doi: 10.1186/1476-4598-9-137 (PMC2898702; doi:10.1186/1476-4598-9-137)
Supplement: Additional file 1 — Figure S1. Effects of D1 on melanoma and neuroblastoma cell proliferation analyzed with drug washout experiments [file 1476-4598-9-137-S1.PPT]

## Slide 1
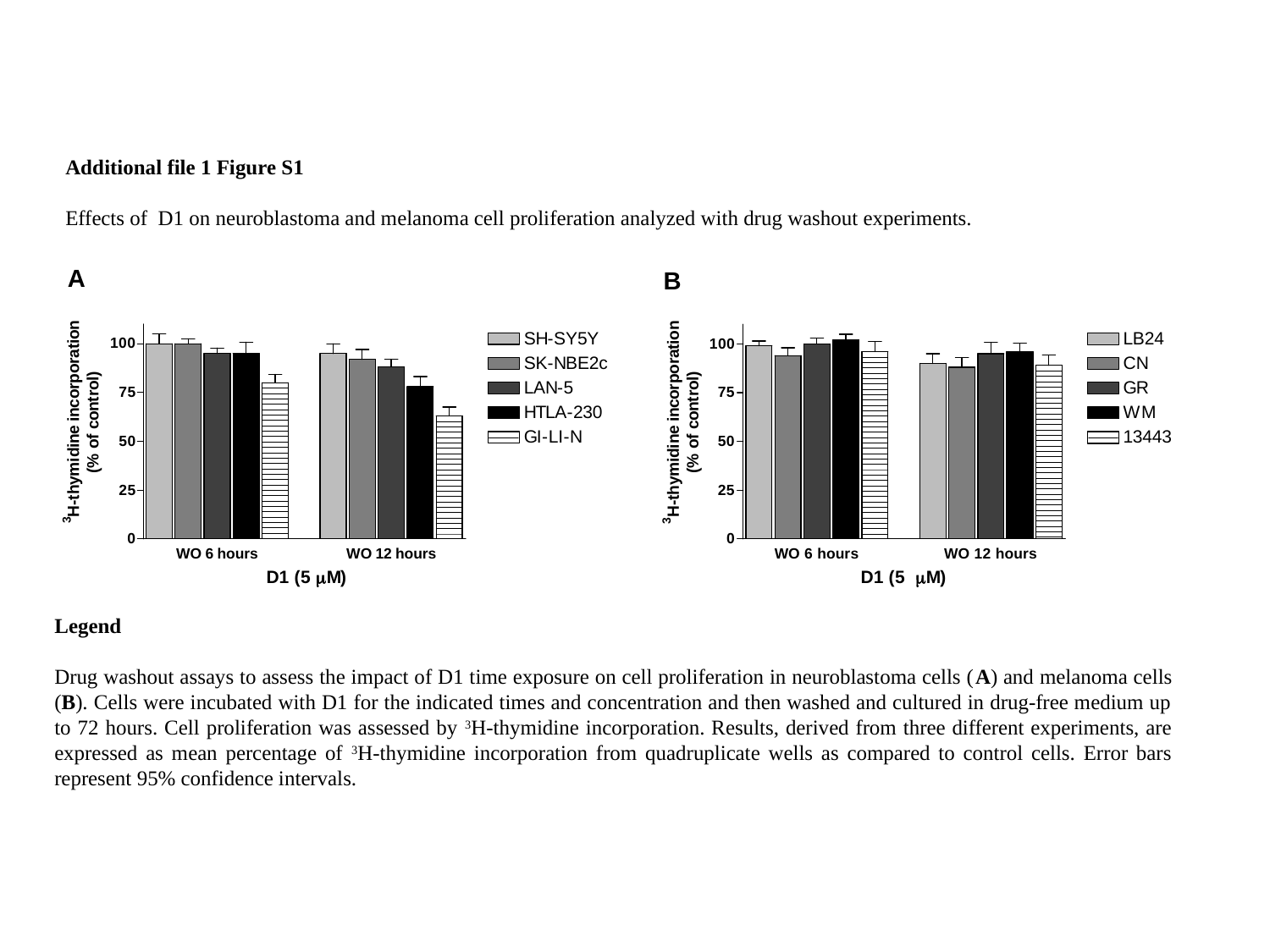

Additional file 1 Figure S1
Effects of D1 on neuroblastoma and melanoma cell proliferation analyzed with drug washout experiments.
A
B
Legend
Drug washout assays to assess the impact of D1 time exposure on cell proliferation in neuroblastoma cells (A) and melanoma cells (B). Cells were incubated with D1 for the indicated times and concentration and then washed and cultured in drug-free medium up to 72 hours. Cell proliferation was assessed by 3H-thymidine incorporation. Results, derived from three different experiments, are expressed as mean percentage of 3H-thymidine incorporation from quadruplicate wells as compared to control cells. Error bars represent 95% confidence intervals.
