# Supplementary material for: Enhanced anti-tumor activity of a new curcumin-related compound against melanoma and neuroblastoma cells
Source: Mol Cancer. 2010 Jun 3;9:137. doi: 10.1186/1476-4598-9-137 (PMC2898702; doi:10.1186/1476-4598-9-137)
Supplement: Additional file 2 — Table S1. Liver and renal toxicity values during the in-vivo therapeutic studies [file 1476-4598-9-137-S2.DOC]

**Additional file 2 Table S1**

Liver and renal toxicity values during the the *in-vivo* therapeutic studies.

Liver and renal toxicity values in LB24 melanoma-bearing mice 2 hours after the first treatment:

|  | AST  IU/L | ALT  IU/L | γGT  IU/L | CRE  mg/dL | BUN  mg/dL |
| --- | --- | --- | --- | --- | --- |
| Control mice | 237 ± 91 | 51 ± 28 | 4 ± 2.2 | 0.4 ± 0.07 | 42 ± 10 |
| D1-treated mice | 178 ± 75 | 51 ± 35 | 4 ± 1 | 0.38 ± 0.05 | 43 ± 5 |
| D6-treated mice | 204 ± 51 | 39 ± 14 | 4 ± 2 | 0.36 ± 0.07 | 44 ± 4 |

and 2 hours after the last treatment:

|  | AST  IU/L | ALT  IU/L | γGT  IU/L | CRE  mg/dL | BUN  mg/dL |
| --- | --- | --- | --- | --- | --- |
| Control mice | 288 ± 90 | 45 ± 21 | 3 ± 2 | 0.35± 0.05 | 36 ± 6 |
| D1-treated mice | 219 ± 45 | 31 ± 17 | 6 ± 2 | 0.39 ± 0.05 | 32 ± 6 |
| D6-treated mice | 209 ± 55 | 42 ± 12 | 4 ± 2 | 0.41 ± 0.05 | 42 ± 6 |

AST= serum glutamic oxaloacetic transaminase; ALT= glutamic-pyruvic transaminase; γGT= gamma-glutamil transpeptidase; CRE= creatinine; BUN= blood urea nitrogen.
