# Supplementary material for: Enhanced anti-tumor activity of a new curcumin-related compound against melanoma and neuroblastoma cells
Source: Mol Cancer. 2010 Jun 3;9:137. doi: 10.1186/1476-4598-9-137 (PMC2898702; doi:10.1186/1476-4598-9-137)
Supplement: Additional file 3 — Figure S2. Effects of D1 and D6 on NFkB expression in neuroblastoma cell line (figure, legend and methods) [file 1476-4598-9-137-S3.PPT]

## Slide 1
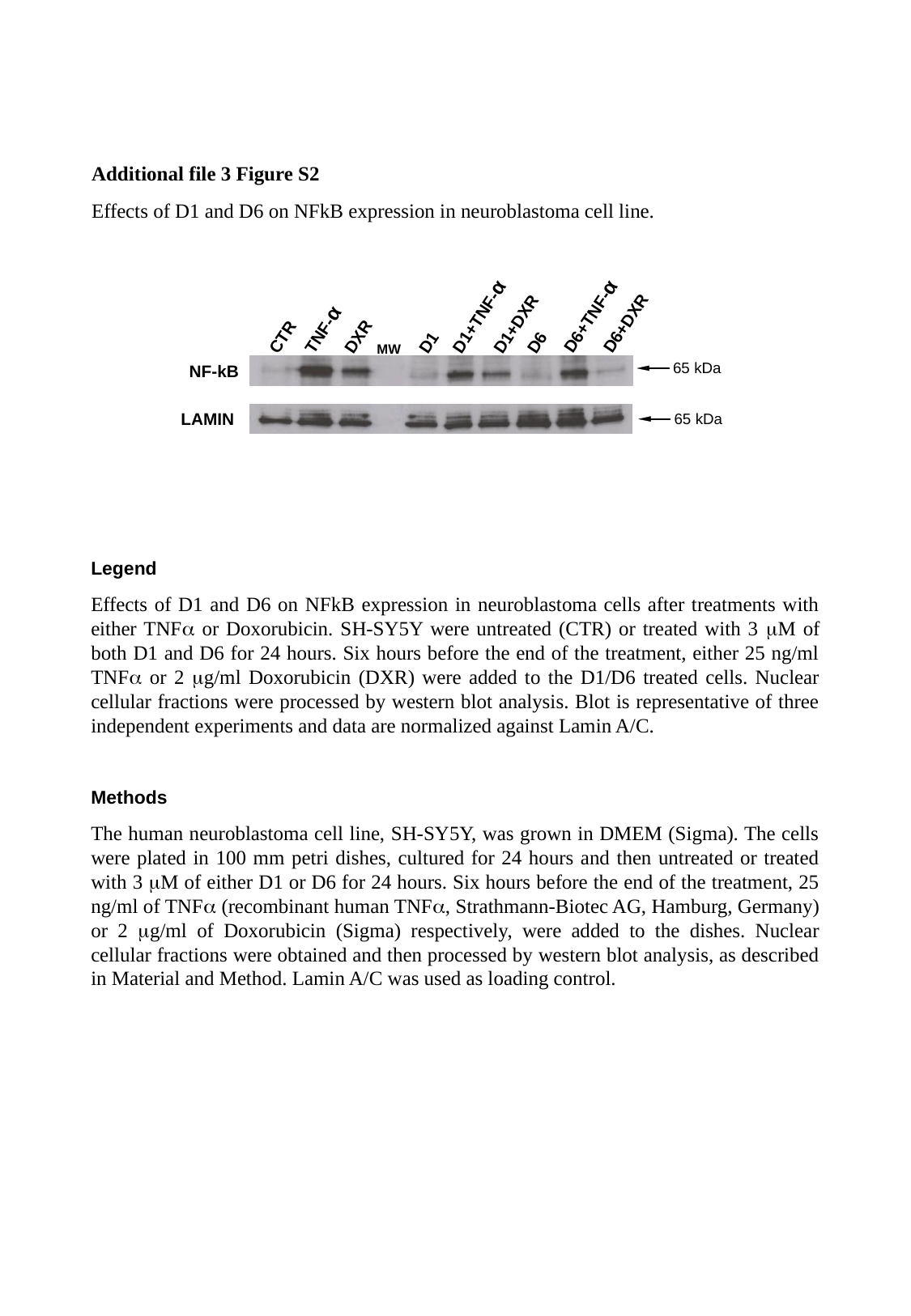

Additional file 3 Figure S2
Effects of D1 and D6 on NFkB expression in neuroblastoma cell line.
D6+TNF-α
D1+TNF-α
D6+DXR
D1+DXR
TNF-α
CTR
D1
D6
DXR
MW
65 kDa
NF-kB
LAMIN
65 kDa
Legend
Effects of D1 and D6 on NFkB expression in neuroblastoma cells after treatments with either TNFα or Doxorubicin. SH-SY5Y were untreated (CTR) or treated with 3 μM of both D1 and D6 for 24 hours. Six hours before the end of the treatment, either 25 ng/ml TNFα or 2 μg/ml Doxorubicin (DXR) were added to the D1/D6 treated cells. Nuclear cellular fractions were processed by western blot analysis. Blot is representative of three independent experiments and data are normalized against Lamin A/C.
Methods
The human neuroblastoma cell line, SH-SY5Y, was grown in DMEM (Sigma). The cells were plated in 100 mm petri dishes, cultured for 24 hours and then untreated or treated with 3 μM of either D1 or D6 for 24 hours. Six hours before the end of the treatment, 25 ng/ml of TNFα (recombinant human TNFα, Strathmann-Biotec AG, Hamburg, Germany) or 2 μg/ml of Doxorubicin (Sigma) respectively, were added to the dishes. Nuclear cellular fractions were obtained and then processed by western blot analysis, as described in Material and Method. Lamin A/C was used as loading control.
